# Updating unanswered questions for stillbirth research: refresh of the UK Stillbirth Priority Setting Partnership

**DOI:** 10.1002/uog.70261

**Published:** 2026-06-21

**Authors:** A. E. P. Heazell, N. Sobolewski, E. Aalai, S. W. Glover, K. Wolffs, C. Storey, the Stillbirth Priority Setting Partnership Steering Group, Kath Abrahams, Karen Burgess, Alicia Burnett, Kate Davies, Amneet Graham, Eva M Loucaides, Subhabrata Mitra, William Simmons, Lucy K Smith, Helen Tebay, Sara Webb, Melissa Whitworth, Ben Wills

**Affiliations:** ^1^ Maternal and Fetal Health Research Centre, Faculty of Biology, Medicine and Health University of Manchester Manchester UK; ^2^ Saint Mary's Hospital Manchester University Hospitals NHS Foundation Trust, Manchester Academic Health Science Centre Manchester UK; ^3^ Library Service Manchester University Hospitals NHS Foundation Trust, Manchester Academic Health Science Centre Manchester UK; ^4^ James Lind Alliance National Institute for Health and Care Research, Evaluation Trials and Studies Coordinating Centre Southampton UK; ^5^ International Rainbow Clinic Network Manchester UK

**Keywords:** biomedical research, fetal death, fetal demise, health priorities, perinatal death, pregnancy outcome, public involvement

## Abstract

**Objectives:**

Despite UK targets to reduce stillbirth, there has been comparatively less research focused on stillbirth than on other pregnancy complications. This study aimed to ensure that future research addresses the most important contemporary questions regarding stillbirth by updating the 2015 UK Stillbirth Priority Setting Partnership (PSP), in accordance with the James Lind Alliance (JLA), in collaboration with over 30 professional and stakeholder organizations.

**Methods:**

The Stillbirth PSP was accepted by the JLA for a refreshed list of priorities in June 2024, and a steering group was formed. A survey was then developed in English to identify potential research questions regarding stillbirth and perinatal death in the UK. The initial survey was open from 3 February 2025 to 7 April 2025, during which professionals and people affected by stillbirth were invited to submit research questions relating to either the causes, impact, prevention or management of stillbirth or pregnancy loss after 20 weeks' gestation. The questionnaire was publicized via social media and by stakeholder organizations. Participants' responses were analyzed, duplicate or out‐of‐scope questions were removed, and indicative questions were formulated from those submitted. Literature searches were carried out in MEDLINE, EMBASE, CINAHL, Cochrane Library and PsychInfo to identify which of the submitted questions had been answered in previously published work. The remaining unanswered research questions were carried forward into a second prioritization survey, which was hosted online from 15 September 2025 to 16 October 2025. The research questions at the highest priority level were determined by consensus at a face‐to‐face workshop in November 2025, involving participants with lived experience and healthcare professionals.

**Results:**

The initial survey received 1261 responses from 525 participants. A further 24 research questions were identified from 10 clinical practice guidelines. Of these 1285 questions, 120 were out of scope. After removing duplicates and combining responses, there were 89 indicative questions. Literature searches determined that 10 questions had been answered previously. The remaining 79 questions were carried forward into the second prioritization survey and were ranked by 441 participants. The top 26 questions were taken to the face‐to‐face workshop, which prioritized 12 research questions by consensus. The prioritized topics for future research included prediction, prevention, understanding of the causes and management of stillbirth.

**Conclusions:**

This updated Stillbirth PSP indicates that researchers should prioritize studies addressing the identified research priorities, because these reflect the most important research questions for those affected by stillbirth and frontline professionals. © 2026 The Author(s). *Ultrasound in Obstetrics & Gynecology* published by John Wiley & Sons Ltd on behalf of International Society of Ultrasound in Obstetrics and Gynecology.

## INTRODUCTION

The UK government set a target in 2015 to reduce the incidence of stillbirth and neonatal death by 50% by 2025, relative to 2013 levels. Although there has been a significant reduction, this target has not yet been achieved and rates of stillbirth and neonatal death in the UK remain higher than in comparable nations, with a significant proportion associated with suboptimal care^1^, ^2^. Although stillbirth and perinatal death are associated with significant economic, medical, psychological and social impacts, they remain under‐researched^3^, ^4^. For example, a literature search for stillbirth in humans, conducted in MEDLINE on 18 October 2025, yielded 17 895 results compared with 54 938 for pre‐eclampsia and 93 497 for preterm labor. Thus, further studies are needed to address key challenges relating to stillbirth and perinatal death.

The initial UK Stillbirth Priority Setting Partnership (PSP) commenced in 2014, with the top 11 research priorities identified by the PSP published in 2015^5^. These research priorities included assessment of fetal growth and the placenta during pregnancy, determining which investigations identify fetuses at risk of stillbirth, describing modifiable factors associated with stillbirth, understanding the causes of stillbirth and why the incidence of stillbirth in the UK is higher than in comparable countries, and determining the most appropriate care after perinatal bereavement and in subsequent pregnancies^5^. In the intervening decade, there have been increasing numbers of published studies and a series of related PSPs on topics including miscarriage^6^, preterm birth^7^, multiple pregnancy^8^, placental pathology^9^ and care of subsequent pregnancy after stillbirth^10^. Whilst some of these PSPs have followed the methodology outlined by the James Lind Alliance (JLA), others, including The Lancet's Stillbirth Series published in 2011^11^ and PSPs on placental histopathology^9^ and care in subsequent pregnancy^10^, restricted involvement to expert healthcare professionals, researchers and advocates, excluding participants with lived experience. However, inclusion of people with lived experience is critical as their involvement significantly alters healthcare and research priorities, making these more patient‐centered.

The objective for this Stillbirth PSP was to update the research priorities established in 2015. To achieve this, we aimed to (i) enable parents, stakeholders and healthcare professionals to identify research priorities, (ii) determine which of these research questions had been answered by prior research and (iii) reach consensus on an updated top 10 list of research priorities.

## METHODS

### PSP process

This article reports the Stillbirth PSP process in line with the REPRISE reporting guideline (Appendix S1)^12^. The Stillbirth PSP process was funded by Tommy's (London, UK) and the lead academic organization was the Maternal and Fetal Health Research Centre, University of Manchester, Manchester, UK. The PSP was undertaken in accordance with the JLA methodology. Previous JLA PSPs have been judged to be service evaluations and therefore do not require research ethics committee review^6^.

The Stillbirth PSP was accepted by the JLA for a refreshed list of priorities in June 2024, and a steering group was formed of 16 researchers with expertise in quantitative and qualitative research in perinatal death and priority setting methodology, representatives of relevant stakeholder organizations (i.e. charities, professional organizations) and people with lived experience (https://stillbirthpsp.org.uk/steering_group.htm). Following discussion within the steering group, it was decided to adopt a broader definition of stillbirth than the UK legal definition. This was for several reasons: (1) the UK is an international outlier for the gestational threshold to define stillbirth (24 weeks' gestation), with most high‐resource countries using gestational thresholds of 20 weeks or lower; (2) the causal pathways and impact of the death of a fetus from 20 + 0 to 23 + 6 weeks' gestation are similar to those for loss later in pregnancy; and (3) some causes of stillbirth may be affected by timing of birth and in such cases, an infant may be born alive but in a critical condition and die shortly afterwards, in which case the psychological impact, need for bereavement support and risks in subsequent pregnancy are similar to those for stillbirth^13^, ^14^. Therefore, the scope of this PSP was stillbirth and late pregnancy loss (after 20 weeks' gestation), including neonates who die shortly after birth (within the first 24 h) in the UK. A protocol was agreed by the steering group and published on 25 November 2024^15^. The target audience of research priorities identified in this updated PSP was researchers, policymakers and research funders in the UK.

In addition to the steering group (which had representation from 11 organizations), other organizations were approached to act as partners in this PSP. Organizations were deemed to be relevant if they represented individuals who had direct or indirect experience of stillbirth or perinatal death, or represented professionals who provided care for parents and families who have experienced stillbirth or perinatal death. Twenty‐two organizations agreed to be partners, resulting in over 30 organizations collaborating on this project, including those represented by members of the steering group.

A survey was developed by consensus among all members of the steering group, in the English language, to identify research questions on stillbirth and perinatal death in the UK. The survey included an explanation about the PSP process and its scope, with an open question enabling participants to record up to 10 research questions of importance to them. Data were also collected on demographic characteristics of the participants, including their role (e.g. parent, family member, obstetrician, midwife), sex, age and ethnicity. Participation in the PSP did not require research ethics approval; however, participants were informed that their submitted questions would be recorded. The survey was open from 3 February 2025 to 7 April 2025. The link to the survey was publicized via social media and by partner organizations. A focus group was conducted for members of the Jewish community in Manchester because our previous public engagement projects noted women in this demographic group engaged rarely with social media. In addition to the submitted research questions, UK clinical practice guidelines relating to pregnancy (National Institute of Health and Care Excellence (NICE), Royal College of Obstetricians and Gynaecologists (RCOG) and Scottish Intercollegiate Guidelines Network (SIGN)) were searched by A.E.P.H to identify further research questions (Appendix S2).

After the closing date for the survey, participants' responses were downloaded into Microsoft Excel (Microsoft Corp., Redmond, WA, USA). Research questions were screened by two investigators (N.S., A.E.P.H.) and removed if they were out of scope, such as questions that addressed earlier pregnancy loss or did not contain a research question. The initial research questions were categorized into topics by two investigators (N.S., A.E.P.H.) and disagreement was resolved by consensus between the steering group members. Research questions were then distributed to pairs of steering group members to formulate indicative research questions. All indicative questions were carefully mapped back to the initial submissions to ensure key components were retained throughout the process. After a list of 89 indicative research questions had been agreed by the steering group, literature searches were carried out in MEDLINE, EMBASE, CINAHL, Cochrane Library and PsychInfo (when relevant) to determine whether the question had been answered by previous publications. The searches were conducted by members of the Manchester Foundation Trust libraries team, under the supervision of two authors (E.A., S.G), and were carried out in July and August 2025. A research question was deemed to be answered if it had been addressed by high‐grade evidence (i.e. systematic review or similar quality of evidence, e.g. national epidemiology report). The results of the literature searches were reviewed by two authors (L.K.S., A.E.P.H.) and research questions that had been answered already were agreed on following discussion with the steering group. The list of questions deemed to be answered already and the relevant references are given in Table S1.

The remaining 79 unanswered research questions were carried forward into the second prioritization survey. Participants were asked to select up to 10 questions that they regarded as the most important. The questionnaire was hosted online and to prevent bias from the placement of questions, the order of the list was randomized. Participants could re‐enter the webpage to alter their selection up until the closing date of the survey. This prioritization survey was open from 15 September 2025 to 16 October 2025 (extended deadline). It was open to all and was publicized using the same approaches as the initial survey, with an additional link sent to participants of the initial survey who had provided their e‐mail address. Respondents were asked only to give their role (e.g. parent, healthcare professional, charity) in this survey. A further focus group was held with members of the Jewish community in Manchester.

After the survey had closed, responses were downloaded into Microsoft Excel. The research questions were ordered from most to least frequently selected within participants' top 10, stratified by respondent group (parent/lived experience, healthcare professional, other). The highest‐ranked unanswered research questions from each group were compared, and only those selected by ≥ 20% of respondents in each group were taken forward to a face‐to‐face workshop. The workshop was held in November 2025 and involved 15 participants with lived experience (including mothers, fathers, siblings and grandparents) who had responded to the initial questionnaire and indicated willingness to participate in the prioritization workshop and 15 healthcare professionals (including obstetricians, midwives, a neonatologist, perinatal pathologists, a counsellor and a community worker). The 30 participants were purposefully selected to ensure a diverse range of perspectives. The workshop ranked the unanswered questions through three rounds of group discussion, with groups consisting of both professionals and people with lived experience, and the top 12 research priorities were identified. Workshop participants were offered travel expenses and payment for their time.

## RESULTS

**Figure 1:**
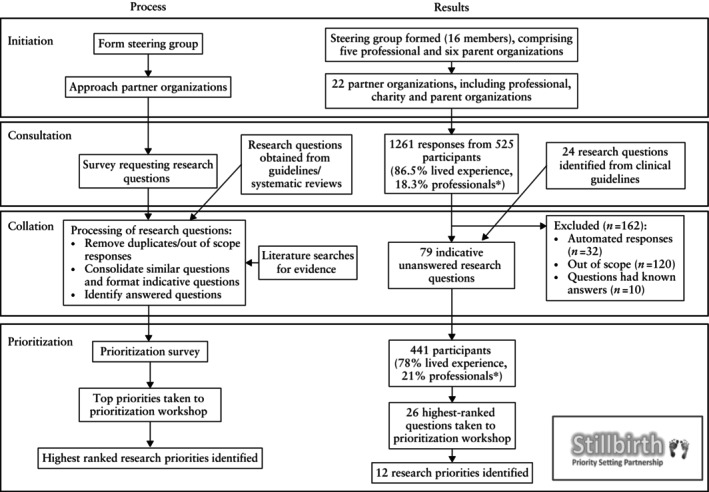
Flowchart demonstrating Stillbirth Priority Setting Partnership process, showing the number of participants who responded to each survey and number of research questions received at each stage. *Professionals include healthcare professionals, charity workers and commercial workers.

The initial survey received responses from 525 participants, and the demographic characteristics of the respondents are presented in Table 1. Overall, 86.48% of respondents were people with lived experience and 18.29% were healthcare professionals, charity workers or commercial workers. Of note, these categories were not mutually exclusive as some respondents identified as health professionals as well as bereaved parents. In addition to midwives and obstetricians, professional respondents included psychologists, counsellors, doulas, pediatricians, perinatal pathologists, physiotherapists and nurses. An overview of the PSP process is given in Figure 1.

**Table 1:** Characteristics of participants who responded to the initial survey to identify research questions on stillbirth and perinatal death (n = 525)

Characteristic	Value
Role*	
Charity worker	13 (2.5)
Commercial worker	2 (0.4)
Midwife	27 (5.1)
Obstetrician	14 (2.7)
Researcher	4 (0.8)
Other professional	36 (6.9)
Parent/family member	423 (80.6)
Friend	31 (5.9)
No response	30 (5.7)
Age	
< 21 years	1 (0.2)
21–25 years	15 (2.9)
26–30 years	52 (9.9)
31–35 years	118 (22.5)
36–40 years	101 (19.2)
41–45 years	100 (19.0)
46–50 years	35 (6.7)
51–55 years	27 (5.1)
56–60 years	16 (3.0)
61–65 years	20 (3.8)
> 65 years	18 (3.4)
No response	22 (4.2)
Ethnicity	
Black African	9 (1.7)
Black Caribbean	10 (1.9)
Chinese	2 (0.4)
Indian	12 (2.3)
Pakistani	4 (0.8)
White British	400 (76.2)
White Irish	17 (3.2)
White other	43 (8.2)
Mixed White and Asian	11 (2.1)
Mixed White and Black Caribbean	3 (0.6)
Other	6 (1.1)
No response	8 (1.5)
Sex	
Female	496 (94.5)
Male	21 (4.0)
Prefer not to say	3 (0.6)
No response	5 (1.0)
Sexual orientation	
Heterosexual	493 (93.9)
Homosexual	6 (1.1)
Bisexual	14 (2.7)
Prefer not to say	5 (1.0)
No response	7 (1.3)

Data are given as *n* (%).

* Role categories were not mutually exclusive.

The initial survey received 1261 research questions from the respondents. In addition to the questions raised by respondents, 24 questions were identified from 10 clinical practice guidelines (Appendix S2). Of these 1285 questions, 120 responses were excluded as they were out of scope, along with 32 automated responses, e.g. irrelevant text from bots. The remaining questions were grouped into 89 indicative questions, following removal of duplicates and consolidation of questions. An example of how the original submitted questions were grouped into themes and then how research questions were subsequently derived is shown in Table S2. Literature searches using appropriate search terms for individual questions found that 10 had been answered previously (Table S1). In the subsequent prioritization survey of these remaining 79 questions, there were 441 participants, of whom 78% were people with lived experience and 21% were healthcare professionals; respondents submitted a total of 4243 votes. Of the 79 unanswered questions ranked in the prioritization questionnaire, the top 26 questions identified as highest ranking for parents, healthcare professionals and those from other backgrounds were taken forward to the face‐to‐face workshop as they were prioritized by ≥ 20% of respondents. The final ranking of the unanswered 26 research questions ranked questions 10–12 with the same arithmetic mean, which were then ranked on geometric mean. The steering group discussed the questions ranked 10–12 and decided that the top 12 research questions would be included in the final list of priorities for stillbirth research, rather than a top 10 (Table 2).

**Table 2:** Ranking of 26 unanswered research questions ranked during face‐to‐face workshop

Rank	Question
1*	How can placental problems be detected (e.g. through extra monitoring), prevented and treated in current and future pregnancies to reduce stillbirth/perinatal death?
2*	Why are babies who are the right size for their gestation still dying from unknown causes, and what tests could help identify those at risk?
3*	How can emerging technologies, including AI and smart watches, help predict and prevent stillbirth and perinatal death by identifying those most at risk using clinical markers, screening tools or new diagnostic tests?
4*	Does variation in individual maternity unit policies lead to disparities in rates of stillbirth and perinatal death and if so, would disseminating best practice and learning from the best performing hospitals reduce stillbirth rates and perinatal death rates?
5*	How can it be ensured that medical professionals listen to the voices and instincts of women and birthing people? Could this reduce stillbirth or perinatal death?
6*	Why are stillbirth rates higher among women and birthing people from certain ethnicities (such as Black and Asian) and socioeconomic backgrounds and what measures can be taken to reduce this inequity?
7*	What is the role of negligence in the current rate of stillbirth and perinatal death?
8*	Would more frequent monitoring (e.g. scans and other tests) during pregnancy help reduce stillbirth/perinatal death?
9*	What causes issues with the umbilical cord, how are individual features or problems with the umbilical cord related to stillbirth/perinatal death and how could these issues be prevented (including through screening, scans etc.)?
10*	What interventions are effective in tackling ethnic and socioeconomic disparities in maternity and bereavement care among Black and ethnic minority communities?
11*	What clinical actions can be taken after a change in fetal movements is reported to help prevent stillbirth/perinatal death?
12*	In the UK, the law currently recognizes stillbirth from 24 weeks of pregnancy. What is the evidence behind this legal threshold, and how might the law be improved to better support families and formally recognize babies who die between 20 + 0 and 23 + 6 weeks of pregnancy?
13	How effective are Doppler measurements of mothers' and babies' blood vessels at planning monitoring of baby, and timing of birth, to prevent stillbirth/perinatal death?
14	How could implementing national screening programs for underlying conditions (such as problems with blood clotting, thyroid problems and autoimmune disorders) in all pregnant women/pregnant people help identify those at increased risk and reduce the incidence of stillbirth and perinatal death?
15	What are the short‐term and long‐term physical, mental, emotional and social impacts of stillbirth or perinatal death on mothers, fathers, siblings and wider family members (including relationships and family dynamics) and how can these family members be best supported, both during and after the loss?
16	What tests can be performed after a baby's death to determine the cause of death and provide information to help prevent stillbirth or perinatal death in future pregnancies, including the identification of previously unrecognized placental problems contributing to unexplained stillbirth or perinatal death?
17	What causes PPROM? How can it be predicted/diagnosed and prevented?
18	What interventions can help prevent stillbirth or perinatal death recurring in future pregnancies?
19	What is the best intervention when PPROM occurs (especially between 20 and 24 weeks of pregnancy) to reduce stillbirth and perinatal death, and how do medical professionals best care for women/pregnant people who are experiencing PPROM?
20	What are the physical and psychological impacts of stillbirth or perinatal death on parents and their future attempts to conceive, and how can those trying to conceive, and those experiencing secondary infertility, be best supported after stillbirth/perinatal death?
21	What modern methods can be developed/used to replace cardiotocography in monitoring fetal health to prevent stillbirth and perinatal death?
22	Why do women/pregnant people often only receive optimum maternity care (in pregnancies) following the death of a baby?
23	How can problems with the umbilical cord best be screened for during late pregnancy to help save babies' lives?
24	Should all placentas be tested after birth to identify abnormalities associated with stillbirth or perinatal death (both from live births and where babies have died)?
25	Would continuity of care in pregnancy and pregnancy after loss reduce stillbirth/perinatal death?
26	How can the time taken for parents to receive postmortem and test results after the death of their baby be reduced, and what are the best strategies to ensure timely return of these results and reviews?

* The top 12 research questions are indicated. AI, artificial intelligence; PPROM, preterm prelabor rupture of membranes.

## DISCUSSION

The findings of this updated Stillbirth PSP provide a consensus‐driven research agenda for stillbirth and perinatal death, ensuring that future investigative efforts address the questions of greatest importance to all stakeholders, including bereaved families and healthcare providers. The results of this PSP have some similarities and some differences to the 2015 Stillbirth PSP^5^. Enduring research gaps relate to unanswered questions around how placental structure and function could be assessed during pregnancy, the most appropriate investigations after perception of reduced fetal movements, what causes stillbirth in appropriately grown fetuses and wider uses of tests, including ultrasound and biochemical assessment and fetal monitoring. Emerging novel research questions in this updated Stillbirth PSP include whether artificial intelligence and smart technologies could be used to prevent stillbirth, reflecting the more recent development of these technologies. In addition, the inclusion of questions about the role of negligence in stillbirth in the UK and the importance of listening to service users could reflect contemporary concerns about accountability, quality of care and the experiences of bereaved families, which have been highlighted in reviews and enquiries of individual maternity services^16^, ^17^.

### Strengths and limitations

This PSP process was strengthened by the increase in the proportion of people with lived experience participating in the survey compared to the initial Stillbirth PSP in 2015. However, despite efforts to include perspectives from groups who have been previously under‐represented in research, these same groups remained under‐represented in our sample relative to their proportion among those affected by stillbirth. For example, 3% of respondents were Asian and 4% were Black, compared with 19% and 11% of stillbirths occurring in these groups, respectively^18^. Furthermore, there may have been a selection bias as social media was used as the primary method of recruitment and the survey was only available in English. Although the final face‐to‐face workshop included a diverse range of participants comprising a wide range of professionals and parents/family members with a range of experiences of perinatal death, constraints on the number of participants meant that not all sociodemographic groups could be represented. A key challenge for standalone projects such as this PSP is that there is limited opportunity to establish relationships with partner organizations to maximize the reach of surveys. Our prior public engagement work with Jewish women gave us additional insights into their community (i.e. that they infrequently use social media), which strengthened our approach to accessing participants from this community. Targeted approaches for other communities could have also helped to increase participation from these groups.

Additionally, maintaining relationships with a range of stakeholder organizations is critical to contribute to a wider research program focused on reducing the impact of stillbirth and perinatal death, of which this PSP is a part. Future studies, particularly those examining outcomes that are more prevalent among non‐English speaking or specific ethnic groups, should allow additional time to engage these communities and be adequately resourced to support translation into relevant languages.

One challenge with using frequency of inclusion of a research question in participants' top 10 selections to identify research priorities is that research questions relating to specific clinical situations (e.g. preterm prelabor rupture of membranes) or smaller demographic groups (e.g. LGBTQIA+ people) may be selected less often, which may underestimate their perceived priority. Consideration should still be given to addressing these unanswered questions, as they are likely of significance to service users in these groups. For example, the 2025 LGBTQIA+ perinatal care PSP reported two questions relating to stillbirth in their top 10 identified research priorities^19^, but as only approximately 4% of the maternal population identify as LGBTQIA+^20^ this may explain why these questions did not achieve sufficient priority to be included in the updated Stillbirth PSP. This emphasizes the importance of checking research priorities in related PSPs to ensure that research in a given field is inclusive.

### Comparison with other PSPs

There have been several PSPs relating to pregnancy conditions and one other PSP specifically relating to stillbirth (not including the 2015 UK PSP)^21^. The Australian Stillbirth PSP was reported in 2025, following a modified JLA process that began in 2020. This exercise utilized workshops to reach different groups of stakeholders between 2020 and 2023, followed up by a prioritization survey of 219 individuals and a face‐to‐face workshop with 26 people. The Australian PSP grouped the 26 prioritized questions into six themes: (i) determine the causes of and pathways that lead to stillbirth; (ii) identify and implement strategies to prevent stillbirth; (iii) build capacity of health services and systems to safely reduce stillbirth rates; (iv) understand and improve the care of families after perinatal loss; (v) ensure culturally sensitive and responsive care for Aboriginal and Torres Strait Islander families to safely reduce stillbirth rates and improve care after perinatal loss; and (vi) ensure culturally sensitive and responsive care for families of migrant and refugee backgrounds to safely reduce stillbirth rates and improve care after perinatal loss^21^. When comparing the themes of individual research questions between the Stillbirth PSPs conducted in Australia^21^ and the UK, there are important similarities, suggesting that these research priorities may be relevant in other high‐resource countries. These questions relate to the understanding of unexplained stillbirth, clinical actions after maternal perception of reduced fetal movements and the role of biomarkers or screening to predict pregnancy complications. It is important to note that both the Australian and UK Stillbirth PSPs identified the need for research to specifically address the disproportionately high stillbirth rates seen in non‐White populations. Research to identify effective interventions to reduce high stillbirth rates in these populations urgently needs to be undertaken as this was first prioritized 10 years ago in the Ending Stillbirth Series, published in *The Lancet* in 2016^22^.

**Figure 2:**
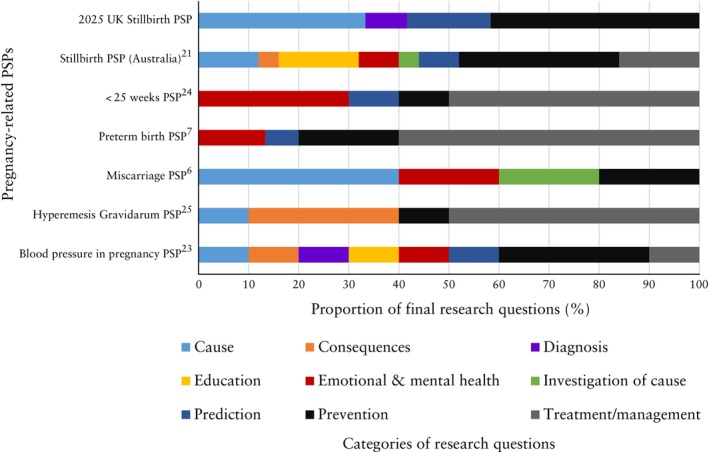
Bar chart showing the proportion of research questions relating to individual themes in seven pregnancy‐related Priority Setting Partnerships (PSPs), including those identified in the present 2025 UK Stillbirth PSP.

Comparison of the research topics prioritized across pregnancy‐related PSPs shows unsurprising variation between them (Figure 2)^6^, ^7^, ^21^, ^23^, ^24^, ^25^. Prevention of the outcome relating to the focus of each PSP was the most common element (10–42% of questions), with the related topic of prediction of the outcome also being a focus (0–17% of questions). Questions relating to the cause were more frequent in Miscarriage and Stillbirth PSPs, emphasizing the high proportion of cases in which the underlying reason for the pregnancy loss is unknown in comparison to preterm birth or hypertensive disorders of pregnancy, for which the etiology is perceived to be better understood. The variation in the topics of prioritized research questions demonstrates that service users and frontline professionals identify a range of issues bespoke to individual clinical scenarios, emphasizing that codevelopment of research questions is critical to identify the most important topics.

### Conclusion

This updated Stillbirth PSP represents a critical step toward ensuring research efforts are aligned with the priorities of those most affected. The previous Stillbirth PSP in 2015 prompted studies that addressed prioritized research questions (e.g. routine ultrasound scans, modifiable risk factors)^26^, ^27^. Resources and funding should be prioritized for studies that address the prioritized questions; critically, funding streams are available to support PSP research priorities. The persistence of certain priorities from the previous PSP is a stark reminder that barriers exist to addressing stillbirth research questions. For example, addressing prediction of stillbirth can be challenging in low‐burden, high‐resource settings, meaning that sample sizes need to be large, requiring multicenter collaborations. Consideration should be given to efficient study designs that generate the highest‐grade evidence possible to inform clinical practice. It is anticipated that acknowledgment of the impact of stillbirth on families and focusing on prioritized research questions will facilitate studies in this area and achieve the desired impact, reducing stillbirth and improving care when a death occurs.

## Collaborators

**Members of the Stillbirth PSP Steering Group:**

**K. Abrahams**, Tommy's, London, UK

**K. Burgess**, Petals, Swaffham Bulbeck, UK

**A. Burnett**, Black Baby Loss Awareness Week, UK

**K. Davies**, Tommy's, London, UK

**A. Graham**, Willow's Rainbow Box, Newcastle upon Tyne, UK

**E. M. Loucaides**, St George's University Hospital NHS Foundation Trust, City St George's University of London, London, UK

**S. Mitra**, Institute for Women's Health, University College London, London, UK

**W. Simmons**, Department of Paediatric Histopathology, Alder Hey Children's NHS Foundation Trust, Liverpool, UK

**L. K. Smith**, Division of Nursing Sciences, Pregnancy and Child Health, School of Healthcare, University of Leicester, Leicester, UK

**H. Tebay**, Miscarriage Association, Wakefield, UK

**S. Webb**, Royal College of Midwives, London, UK

**M. Whitworth**, Saint Mary's Hospital, Manchester University Hospitals NHS Foundation Trust, Manchester Academic Health Science Centre, Manchester, UK

**B. Wills**, Sands, London, UK

## Supporting information


**Appendix S1** REPRISE reporting guide.

**Appendix S2** Research questions identified from clinical guidelines.

**Table S1** Research questions answered already by high‐quality evidence.

**Table S2** Example of how 11 original questions submitted by participants were grouped into two research themes and then developed into a single research question.

## Data Availability

The data that support the findings of this study are available from the corresponding author upon reasonable request.

## References

[1] Draper ES , Kurinczuk JJ , Kenyon S , MBRRACE‐UK. obo . MBRRACE‐UK perinatal confidential enquiry: term, singleton, normally formed, antepartum stillbirth. Leicester: the infant mortality and morbidty studies. Department of Health Sciences, University of Leicester; 2015.

[2] Draper ES , Kurinczuk JJ , Kenyon S , MBRRACE‐UK. obo . MBRRACE‐UK perinatal confidential enquiry: term, singleton, intrapartum stillbirth and intrapartum‐related neonatal death. Leicester: the infant mortality and morbidty studies. Department of Health Sciences, University of Leicester; 2017.

[3] Heazell AEP , Siassakos D , Blencowe H , et al. Stillbirths: economic and psychosocial consequences. Lancet. 2016;387(10018):604‐616. 26794073 10.1016/S0140-6736(15)00836-3

[4] Agravat P , Loucaides EM , Kumar MB , et al. Research funding for newborn health and stillbirths, 2011–20: a systematic analysis of levels and trends. Lancet Glob Health. 2023;11(11):e1794‐e1804. 37858589 10.1016/S2214-109X(23)00379-0 PMC10603613

[5] Heazell AE , Whitworth MK , Whitcombe J , et al. Research priorities for stillbirth: process overview and results from UK Stillbirth Priority Setting Partnership. Ultrasound Obstet Gynecol. 2015;46(6):641‐647. 26336941 10.1002/uog.15738

[6] Prior M , Bagness C , Brewin J , et al. Priorities for research in miscarriage: a priority setting partnership between people affected by miscarriage and professionals following the James Lind Alliance methodology. BMJ Open. 2017;7(8):e016571. 10.1136/bmjopen-2017-016571 PMC5629698 28838896

[7] Oliver S , Uhm S , Duley L , et al. Top research priorities for preterm birth: results of a prioritisation partnership between people affected by preterm birth and healthcare professionals. BMC Pregnancy Childbirth. 2019;19(1):528. 31888523 10.1186/s12884-019-2654-3 PMC6938013

[8] Lam JR , Liu B , Bhate R , et al. Research priorities for the future health of multiples and their families: the global twins and multiples priority setting partnership. Ultrasound Obstet Gynecol. 2019;54(6):715‐721. 31600847 10.1002/uog.20858

[9] Marijnen MC , Bugel MI , Khong TY , et al. Priority setting partnership on placental pathology: consensus recommendations for placental research. Placenta. 2025;160:67‐72. 39778255 10.1016/j.placenta.2024.12.020

[10] Wojcieszek AM , Heazell AE , Middleton P , et al. Research priorities and potential methodologies to inform care in subsequent pregnancies following stillbirth: a web‐based survey of healthcare professionals, researchers and advocates. BMJ Open. 2019;9(6):e028735. 10.1136/bmjopen-2018-028735 PMC6596997 31230027

[11] Flenady V , Middleton P , Smith GC , et al. Stillbirths: the way forward in high‐income countries. Lancet. 2011;377(9778):1703‐1717. 21496907 10.1016/S0140-6736(11)60064-0

[12] Tong A , Synnot A , Crowe S , et al. Reporting guideline for priority setting of health research (REPRISE). BMC Med Res Methodol. 2019;19(1):243. 31883517 10.1186/s12874-019-0889-3 PMC6935471

[13] Smith LK , Dickens J , Bender Atik R , et al. Parents' experiences of care following the loss of a baby at the margins between miscarriage, stillbirth and neonatal death: a UK qualitative study. BJOG. 2020;127(7):868‐874. 31976622 10.1111/1471-0528.16113 PMC7383869

[14] Woolner AMF , Shestopaloff K , Heazell AEP . Pregnancy outcomes after second trimester pregnancy loss and termination for medical reasons before 24 weeks: a historical cohort study [PASTeL‐2]. BJOG. 2026;133(6):1200‐1212. 41582556 10.1111/1471-0528.70161 PMC13040414

[15] Stillbirth PSP Steering Group . Stillbirth Refresh PSP protocol. James Lind Alliance; 2024 Accessed 3 March 2026 https://www.jla.nihr.ac.uk/documents/stillbirth‐refresh‐psp‐protocol.

[16] Kirkup B . The report of the morecambe bay investigation. Morecambe Bay Investigation; 2015.

[17] Ockenden D . Findings, conclusions and essential actions from the independent review of maternity services at the Shrewsbury and Telford Hospital NHS Trust. UK Government; 2022.

[18] Gallimore ID , Matthews RJ , Page GL , et al. MBRRACE‐UK perinatal mortality surveillance report, UK perinatal deaths for births from January to December 2023. Leicester: the infant mortality and morbidity studies. Department of Health Sciences, University of Leicester; 2025.

[19] Matheson A , Bainbridge A , Kiama Zuri E , et al. LGBTQIA+ Perinatal Care – Top 10 Research Priorities. James Lind Alliance; 2025 Accessed 9 January 2026 https://www.jla.nihr.ac.uk/priority‐setting‐partnerships/lgbtqia‐perinatal‐care#tab‐top‐10‐priorities.

[20] Greenfield M , Darwin Z . LGBTQ+ new and expectant parents' experiences of perinatal services during the UK's first COVID‐19 lockdown. Birth. 2024;51(1):134‐143. 37803934 10.1111/birt.12780

[21] Tindal K , Boyle F , Andrews C , et al. Research priorities for stillbirth in Australia: outcomes of a national priority setting partnership. BMC Pregnancy Childbirth. 2025;26(1):94. 41449344 10.1186/s12884-025-08552-6 PMC12849368

[22] de Bernis L , Kinney MV , Stones W , et al. Stillbirths: ending preventable deaths by 2030. Lancet. 2016;387(10019):703‐716. 26794079 10.1016/S0140-6736(15)00954-X

[23] Ho A , Webster L , Bowen L , et al. Research priorities for pregnancy hypertension: a UK priority setting partnership with the James Lind Alliance. BMJ Open. 2020;10(7):e036347. 10.1136/bmjopen-2019-036347 PMC7365422 32665388

[24] Peart S , Ray O , Galletta L , et al. Research priorities for the most premature babies born < 25 weeks' gestation: results of an international priority setting partnership. Arch Dis Child Fetal Neonatal Ed. 2025;110(6):556‐563. 39988355 10.1136/archdischild-2024-328133 PMC12573321

[25] Dean CR , Bierma H , Clarke R , et al. A patient‐clinician James Lind Alliance partnership to identify research priorities for hyperemesis gravidarum. BMJ Open. 2021;11(1):e041254. 10.1136/bmjopen-2020-041254 PMC7813320 33452191

[26] Smith GC , Moraitis AA , Wastlund D , et al. Universal late pregnancy ultrasound screening to predict adverse outcomes in nulliparous women: a systematic review and cost‐effectiveness analysis. Health Technol Assess. 2021;25(15):1‐190. 10.3310/hta25150 PMC7958245 33656977

[27] Cronin RS , Li M , Thompson JMD , et al. An individual participant data meta‐analysis of maternal going‐to‐sleep position, interactions with fetal vulnerability, and the risk of late stillbirth. EClinicalMedicine. 2019;10:49‐57. 31193832 10.1016/j.eclinm.2019.03.014 PMC6543252

