# Supplementary material for: Updating unanswered questions for stillbirth research: refresh of the UK Stillbirth Priority Setting Partnership
Source: Ultrasound Obstet Gynecol. 2026 Jun 21;68(2):248–55. doi: 10.1002/uog.70261 (PMC13432989; doi:10.1002/uog.70261)
Supplement: Supplementary file 1 — Appendix S1 REPRISE reporting guide. [file UOG-68-248-s001.docx]

| **No** | **Item** | **Descriptor and/or examples** | **Location in Manuscript** |
| --- | --- | --- | --- |
| A | Context and scope |  |  |
| 1 | Define geographical scope | Global, regional, national, city, local area, institutional/organizational level, health service | Introduction is UK-focussed, abstract, methods and line 135 clearly state UK. |
| 2 | Define health area, field, focus | Disease or condition specific, interventions, healthcare delivery, health system | Title, Abstract and Introduction focus on stillbirth |
| 3 | Define the intended beneficiaries | This may include the general population or a specific population based on demographic (age, gender), clinical (disease, condition), or other characteristics who may benefit from the research | Focussed on those who have experienced a stillbirth or perinatal death |
| 4 | Define the target audience of the priorities | Policy makers, funders, researchers, industry or others who have the potential to implement the priorities identified | Lines 136-137 |
| 5 | Identify the research area | Public health, health services research, clinical research, basic science | Title, Abstract and Introduction focus on stillbirth |
| 6 | Identify the type of research questions | Etiology, diagnosis, prevention, treatment (interventions), prognosis, health services, psychosocial, behavioral and social science, economic evaluation, implementation; this may not be pre-defined | No limit on research questions. |
| 7 | Define the time frame | Interim, short-term, long-term priorities, plans to revise and update | This was a refreshed PSP (line 121) |
| B | Governance and team |  |  |
| 8 | Describe the selection and structure of the leadership and management team | Those responsible for initiating, developing, and guiding the process for priority setting, and examples of structures include; Steering Committee, Advisory Group, Technical Experts | Lines 121-123 |
| 9 | Describe the characteristics of the team | Stakeholder group or role, institutional affiliations, country or region, demographics (e.g. age sex), discipline, experience, expertise | Link to website, Line 124 |
| 10 | Describe any training or experience relevant to conducting priority setting | Consultants or advisors, members with experience or skills relevant to the conducting priority-setting e.g. qualitative methods, surveys, facilitation | Lines 122-123 |
| C | Framework for priority setting |  |  |
| 11 | State the framework used (if any) | James Lind Alliance, COHRED, CHNRI, Dialogue Model, no framework (general research priority setting) | Lines 118-119 |
| D | Stakeholders or participants |  |  |
| 12 | Define the inclusion criteria for stakeholders involved in priority-setting | Patients, caregivers, general community, health professionals, researchers, policy makers, non-governmental organizations, government, industry; specific groups including vulnerable and marginalized populations | Lines 138-140 |
| 13 | State the strategy or method for identifying and engaging stakeholders | Partnership with organizations, social media, recruitment through hospitals | Lines 140-142 |
| 14 | Indicate the number of participants and/or organizations involved | Number of individuals and organizations, include number by stakeholder group | Line 144 |
| 15 | Describe the characteristics of stakeholders | Stakeholder group, demographic characteristics, areas of interest and expertise, discipline, affiliations | Lines 140-142 |
| 16 | State if reimbursement for participation was provided | Cash, vouchers, certificates, acknowledgement; what purpose e.g. travel, accommodation, honorarium | Lines 195-196 |
| E | Identification and collection of research priorities |  |  |
| 17 | Describe methods for collecting initial priorities | Methods e.g. Delphi survey, surveys, nominal group technique, interviews, focus groups, meetings, workshops; prioritization e.g. voting, ranking; mode e.g. face-to-face, online; may be informed by evidence e.g. systematic reviews, reviews of guidelines/other documents, health technology assessment | Lines 143-148 |
| 18 | Describe methods for collating and categorizing priorities | Taxonomy or other framework used to organize, summarise, and aggregate topics or questions | Lines 158-161 |
| 19 | Describe methods and reasons for modifying (removing, adding, reframing) priorities | Based on scope, clarity, definition, duplication, other criteria | Lines 161-165 |
| 20 | Describe methods for refining or translating priorities into research topics or questions | Reviewed by Steering Committee or project team | Lines 161-165 |
| 21 | Describe methods for checking whether research questions or topics have been answered | Systematic reviews, evidence mapping, consultation with experts | Lines 166-172 |
| 22 | Describe number of research questions or topics | Number of priorities at each stage of the process | Figure 1, Lines 205-219 |
| F | Prioritization of research topics/questions |  |  |
| 23 | Describe methods and criteria for prioritizing research topics or questions | Methods e.g. Delphi survey, surveys, nominal group technique, interviews, focus groups, meetings, workshops;  Prioritization e.g. voting, ranking;  Mode e.g. face-to-face, online;  Criteria e.g. need, feasibility, novelty, equity | Lines 174-182 |
| 24 | State the method or threshold for excluding research topics/questions | Thresholds for ranking scores, proportions, votes; other criteria | Lines 183-188 |
| G | Output |  |  |
| 25 | State the approach to formulating the research priorities | Area, topic, questions, PICO (population, intervention, comparator, outcome) | Lines 161-162 |
| H | Evaluation and feedback |  |  |
| 26 | Describe how the process of prioritization was evaluated | Survey, workshop | Lines 188-194 |
| 27 | Describe how priorities were fed back to stakeholders and/or to the public; and how feedback (if received) was addressed and integrated | Public meetings or workshop, newsletters, website, email, online presentations | By this report and the stillbirth PSP website and funders website |
| I | Implementation |  |  |
| 28 | Outline the strategy or action plans for implementing priorities | Communication with target audience, via policies and funding | Lines 317-326 |
| 29 | Describe plans, strategies, or suggestions to evaluate impact | Integration in decision-making, funding allocation, review of relevant documents | Lines 313-314 |
| J | Funding and conflict of interest |  |  |
| 30 | State sources of funding | Name sources of funding for the priority-setting exercise; if relevant include the budget and/or cost | Lines 116-117 |
| 31 | Declare any conflicts or competing interests | State any conflicts of interest that may be at an individual level and/or at a contextual level (e.g. political issues, controversies) that may affect the process, output or implementation. | None declared |
