# Supplementary material for: Updating unanswered questions for stillbirth research: refresh of the UK Stillbirth Priority Setting Partnership
Source: Ultrasound Obstet Gynecol. 2026 Jun 21;68(2):248–55. doi: 10.1002/uog.70261 (PMC13432989; doi:10.1002/uog.70261)
Supplement: Supplementary file 2 — Appendix S2 Research questions identified from clinical guidelines. [file UOG-68-248-s003.docx]

**Appendix S2 - Research questions identified from clinical guidelines**

Care of late intrauterine fetal death and stillbirth, RCOG Green top Guideline No. 55, October 2024

1. Effectiveness of stillbirth care guidelines and the bereavement care pathway on core outcomes (core outcome set for stillbirth care)
2. Feasibility and utility of non-invasive and minimally invasive post-mortem examination of the baby.
3. Impact of models of care in subsequent pregnancies after late IUFD.
4. Induction of labour to prevent adverse outcome in subsequent pregnancies after late IUFD.
5. Association between microbiome and pregnancy loss including late IUFD.
6. Effectiveness of bereavement counselling or other interventions post late IUFD.
7. Optimal methods of induction for women with late IUFD.
8. Validation and clinical evaluation of risk prediction models for late IUFD

Vasa Praevia: Diagnosis and Management RCOG Green-top Guideline No. 27b, September 2018

1. National and regional epidemiological data are needed to define a relevant high-risk population and the cost-effectiveness of screening for vasa praevia on service provision.

Antepartum haemorrhage, RCOG, 2011

1. Randomised controlled trials of sufficient power are required to assess interventions (for example diet, vitamin supplements and antithrombotic therapy) to prevent placental abruption.
2. Studies are required to determine the optimum timing of delivery in women presenting with unexplained APH and no associated maternal and/or fetal compromise.

Investigation and Care of a Small-for-Gestational-Age Fetus and a Growth Restricted Fetus (Green-top Guideline No. 31), May 2024

1. The use of biomarkers as a universal screening test to predict adverse pregnancy outcomes including cost effective analysis.
2. Longitudinal studies of fetuses, whose birthweight falls within the normal range but who are suspected of abnormal fetal growth, should address whether additional measures of fetal growth e.g. ultrasound biometry and velocity, Doppler assessment and biomarkers can help determine those at risk of adverse outcome.
3. Further research into routine third trimester USS assessing a package of care (diagnostic accuracy of test and management pathways for screen positive and negative women) considering a range of outcomes are required.
4. Further research into interventions, including pharmacological treatments, for FGR once diagnosed to reduce the risk of adverse outcome.

Care of Women Presenting with Suspected Preterm Prelabour Rupture of Membranes from 24+0 Weeks of Gestation, RCOG Green-top Guideline No. 73, June 2019

1. Methods to monitor the fetus following PPROM require further investigation.

Intrahepatic cholestasis of pregnancy, Green-top Guideline No. 43 June 2022

1. In women with ICP, which maternal or fetal prognostic tools and/or monitoring modalities predict adverse perinatal outcome (including preterm birth and stillbirth)?
2. What is the ongoing risk of adverse pregnancy outcome in women whose bile acid concentrations normalise?

Reduced Fetal Movements, Greentop Guideline 57, Draft 4, April 2025

1. Determine whether other tests of fetal wellbeing (e.g. cerebroplacental ratio, umbilical artery Doppler) or placental compromise (e.g. Placental Growth Factor) identify fetal compromise in women presenting with reduced fetal movements.

Antenatal care, NICE guideline, Reference number:NG201, August 2021

1. Models of antenatal care; What is the clinical and cost effectiveness of different models of antenatal care with varying numbers and times of appointment, and should different models be used for groups at risk of worse outcomes?

Inducing labour - NICE guideline Reference number:NG207, November 2021

1. Prevention of prolonged pregnancy; At what gestational age should induction of labour be offered in the subgroups of women who may be more likely to experience adverse outcomes if pregnancy continues? [2021]
2. Prevention of prolonged pregnancy; Based on individual patient data meta-analysis, what is the optimal timing of induction of labour? [2021]
3. Intrauterine fetal death after previous caesarean birth; How should labour be induced in women with intrauterine fetal death who have had a previous caesarean birth, and who choose to be induced? [2021]

Preterm labour and birth, NICE guideline Reference number:NG25, Published: November 2015, Last updated: 2022

1. Emergency cervical cerclage; What is the clinical effectiveness of emergency cerclage in improving outcomes for women at risk of preterm birth? [2015]
