# Supplementary material for: Updating unanswered questions for stillbirth research: refresh of the UK Stillbirth Priority Setting Partnership
Source: Ultrasound Obstet Gynecol. 2026 Jun 21;68(2):248–55. doi: 10.1002/uog.70261 (PMC13432989; doi:10.1002/uog.70261)
Supplement: Supplementary file 3 — Table S1 Research questions answered already by high‐quality evidence. [file UOG-68-248-s004.docx]

**Table S1** – Research questions answered already by high-quality evidence (systematic review level or equivalent).

| **Answered Question** | **Research Question** | **Published studies** |
| --- | --- | --- |
| 1 | What impact does maternal age have on stillbirth, and perinatal death, rates? | Lean et al. *PLoS One* 2017;12(10):e0186287  <https://doi.org/10.1371/journal.pone.0186287>  Leader et al. 2018;40(9):1208-1218 *JOGC*  <https://doi.org/10.1016/j.jogc.2017.10.027> |
| 2 | What is the relationship between first pregnancies and the risk of stillbirth or perinatal death? | Townsend et al. *BJOG* 2021;128(2):238-250.  <https://doi.org/10.1111/1471-0528.16510> |
| 3 | What role does, or could, aspirin play in preventing stillbirth/perinatal death? | Duley et al. *Cochrane Database Syst Rev* 2019;(10):CD004659. <https://doi.org/10.1002/14651858.CD004659.pub3>  Henderson et al. *JAMA* 2021;326;(12):1192-1206.  <https://doi.org/10.1001/jama.2021.8551>  Man et al. *Eur J Obstet Gynecol Reprod Biol* 2021;262:105-112.  <https://doi.org/10.1016/j.ejogrb.2021.05.017> |
| 4 | Does progesterone reduce the risk of stillbirth or perinatal death and if yes which women/pregnant people should be offered it? | Care et al. *BMJ* 2022;376:e064547  <https://doi.org/10.1136/bmj-2021-064547>  Jarde et al. *BJOG* 2019;126(5):556-567  <https://doi.org/10.1111/1471-0528.15566>  Conde-Agudelo et al. *Am J Obstet Gynecol* 2023;229(6):599-616.e3.  <https://doi.org/10.1016/j.ajog.2023.05.010> |
| 5 | What is the relationship between fertility issues, previous miscarriage, and stillbirth/perinatal death? | Sarmon et al. *Fertil Steril*. 2021 Sep;116(3):784-792.  <https://doi.org/10.1016/j.fertnstert.2021.04.007> |
| 6 | What support could employers offer parents, after stillbirth/perinatal death? | Meunier et al. *Work* 2021;69(2):411-421.  <https://doi.org/10.3233/WOR-213487>  Schoonover et al. *J Loss Trauma* 2023;28(4):348-363  <https://doi.org/10.1080/15325024.2022.2122221> |
| 7 | What are the physical and psychological impacts of stillbirth or perinatal death on parents, in subsequent pregnancies? | Lamont et al. *BMJ* 2015;350:h3080  <https://doi.org/10.1136/bmj.h3080>  Mills et al. *BJOG* 2014;121(8):943-50.  <https://doi.org/10.1111/1471-0528.12656> |
| 8 | Are stillbirth rates in the UK decreasing? | MBBRACE Perinatal Surveillance Report  <https://www.npeu.ox.ac.uk/mbrrace-uk/reports/perinatal-mortality-surveillance/perinatal-surveillance-2023> |
| 9 | What proportion of stillbirths are due to a placental cause? | Kumsa et al. 2024 *Front Med (Lausanne)* 2024:11:1434380.  <https://doi.org/10.3389/fmed.2024.1434380> |
| 10 | Can antibodies to red blood cells be linked to stillbirth? | De Winter et al. *BMC Pregnancy and Childbirth* 2023;23(1):12.  <https://doi.org/10.1186/s12884-022-05329-z>  Prescott et al. 2023 *Hematology, Transfusion and Cell Therapy* 46(3):289-299. <https://doi.org/10.1016/j.htct.2023.07.013> |
