# Supplementary material for: Updating unanswered questions for stillbirth research: refresh of the UK Stillbirth Priority Setting Partnership
Source: Ultrasound Obstet Gynecol. 2026 Jun 21;68(2):248–55. doi: 10.1002/uog.70261 (PMC13432989; doi:10.1002/uog.70261)
Supplement: Supplementary file 4 — Table S2 Example of how 11 original questions submitted by participants were grouped into two research themes and then developed into a single research question. [file UOG-68-248-s002.docx]

**Table S2** – Example of how 11 original questions submitted by participants were grouped into two research themes and then into a single derived research question.

| **Original question submitted** | **Subtheme** | **Derived research question** |
| --- | --- | --- |
| How likely is it to have another stillbirth? | Recurrence of stillbirth | What is the risk of stillbirth or perinatal death recurring in future pregnancies, and what interventions can help prevent such recurrence? |
| Why does it reoccur in some people 2 to 3 times? | Recurrence of stillbirth |  |
| Does still birth cause further pregnancy complications in further pregnancies? | Recurrence of stillbirth |  |
| Is there anything parents can do to help in future pregnancies after experiencing a stillbirth to minimise future risk? | Prevent recurrence of SB |  |
| What can be done to prevent stillbirths/stillbirths in subsequent pregnancies? | Prevent recurrence of SB |  |
| What can health professionals do in order to help prevent another stillbirth in pregnancy after loss? | Prevent recurrence of SB |  |
| Likelihood of recurrent full term still birth? | Recurrence of stillbirth |  |
| What are the chances of having another stillbirth after a normal pregnancy that ended in a still birth? | Recurrence of stillbirth |  |
| What the chances are of another stillbirth and the risks of trying again? | Recurrence of stillbirth |  |
| What likelihood miscarriage or stillbirth is to reoccur based on in-depth medical investigations? | Recurrence of stillbirth |  |
| How prevalent consecutive still births are? | Recurrence of stillbirth |  |
